# Diabetic Kidney Disease in Post-Kidney Transplant Patients

**DOI:** 10.3390/jcm13030793

**Published:** 2024-01-30

**Authors:** Ngoc-Yen T. Pham, Diego Cruz, Luis Madera-Marin, Raja Ravender, Pablo Garcia

**Affiliations:** 1 Division of Nephrology, University of New Mexico School of Medicine, Albuquerque, NM 87106, USA; 2 Hospital General San Juan de Dios, Guatemala City 01001, Guatemala; diadcruz@gmail.com

**Keywords:** PTDM, kidney transplant, diabetic nephropathy, diabetes

## Abstract

Post-transplant diabetes mellitus (PTDM) is a common occurrence in post-kidney transplantation and is associated with greater mortality, allograft failure, and increased risk of infections. The primary goal in the management of PTDM is to achieve glycemic control to minimize the risk of complications while balancing the need for immunosuppression to maintain the health of the transplanted kidney. This review summarizes the effects of maintenance immunosuppression and therapeutic options among kidney transplant recipients. Patients with PTDM are at increased risk of diabetic kidney disease development; therefore, in this review, we focus on evidence supporting the use of novel antidiabetic agents and discuss their benefits and potential side effects in detail.

## 1. Introduction

Kidney transplantation is the preferred therapy for patients with end-stage kidney disease (ESKD). Kidney transplantation prolongs and improves quality of life compared with renal replacement therapy [1,2,3]. While this procedure enhances recipients’ quality of life, it is also associated with multiple complications, such as infections [4], malignancy [5], cardiovascular disease [6], and post-transplant diabetes mellitus [7].

Hyperglycemia is very common after kidney transplantation and is a risk factor for subsequent post-transplantation diabetes mellitus (PTDM) [8,9]. PTDM is defined as newly diagnosed diabetes mellitus in the post-transplantation setting—whether it was present but undetected prior to transplantation or not. PTDM is diagnosed in clinically stable patients without transient post-transplant hyperglycemia [10]. Around one-third of post-transplant patients develop PTDM [11]. PTDM is associated with an increased risk of cardiovascular mortality [12], graft failure, and infections [13]. A meta-analysis that included fourteen retrospective studies revealed an increase in the risk of all-cause mortality in patients with PTDM compared to nondiabetics, in addition to a significant increase in the risk of graft failure compared to nondiabetics [14].

PTDM is distinct from other types of diabetes in terms of its pathophysiology and risk factors. While the exact pathophysiologic mechanisms behind PTDM are not fully understood, the consensus is that it is a combination of pancreatic β-cell dysfunction in the presence of insulin resistance. Immunosuppressive medications are also implicated as a risk factor for PTDM [15]. These immunosuppressive medications are essential to prevent organ rejection in transplant recipients by suppressing the immune system. However, they can also lead to insulin resistance and impaired insulin secretion, eventually resulting in PTDM.

PTDM can significantly impact the overall health and wellbeing of the recipient and requires careful management to ensure the long-term success of the transplanted kidney and to prevent post-transplant diabetic kidney disease. A study examining post-transplant allograft biopsies found that up to 52 percent of diabetic transplant recipients have pathological evidence of diabetic kidney disease at 10 years after transplantation [16]. PTDM care involves a broad range of healthcare providers, including transplant coordinators, primary care providers, pharmacists, and specialists. This review summarizes the mechanisms behind PTDM and its risk factors, the effects of immunosuppression medications, and the latest advancements in its treatment and prevention.

## 2. Pathogenesis of Post-Transplant Diabetes Mellitus

The main underlying pathophysiological mechanism of PTDM is pancreatic β-cell dysfunction [17] in the setting of insulin resistance [18]. Preexisting diabetes risk factors such as obesity, advancing age, and sedentarism can contribute to worsening alterations in insulin sensitivity and β-cell function that could lead to PTDM [19]. A study comparing glycemic metabolic profiles in 1064 kidney allograft recipients versus 1357 nontransplant patients provided evidence to support β-cell dysfunction as the primary defect in PTDM [17]. In addition, in patients with kidney disease, there is a decreased insulin requirement when their renal function deteriorates due to the prolongation of insulin clearance, leading to decreased insulin requirement [20]; this effect is reversed after transplantation, causing an increase in insulin clearance by a functioning allograft, thus leading to difficulty in controlling hyperglycemia and PTDM. Immunosuppression is the major modifiable risk factor for the development of PTDM [15,19].

## 3. Diagnostic Approach for PTDM

There are no specific tests for post-kidney transplant patients; thus, we can only rely on tests utilized in the nontransplant population. As highlighted in the PTDM consensus by Sharif et al., the oral glucose tolerance test (OGTT) is considered the gold standard for PTDM diagnosis [10]. This statement is backed by scientific evidence; in a study including 646 post-transplant recipients who had a fasting glucose test, Sharif et al. looked at those with fasting glucose between 5.6 and 6.9 mmol/L on two separate occasions. This study found that more than 50% of the patients had abnormal glucose metabolism, including 10% with new-onset diabetes. Therefore, this confirms that fasting glucose underestimates the prevalence of new-onset diabetes [21]. Another advantage of OGTT is to allow the diagnosis of impaired glucose tolerance, which is a risk factor for PTDM.

Hemoglobin A1c (HbA1c) could be used to recognize PTDM when elevated. The consensus on PTDM is to recommend being careful with bA1c use because it is unreliable in the presence of anemia or variable allograft function. HbA1c 5.7–6.4% or higher in the early period warrants the need to follow up with a recognized diagnostic test, and HbA1c greater than 6.5% is unlikely to be a false positive [10].

In summary, the PTDM consensus is to recommend using post-prandial glucose monitoring and HbA1 to raise suspicion and use the OGTT to confirm the diagnosis [10]. This approach is valid; however, OGTT is not widely used in clinical practice because it is impractical and time-consuming. Further research is needed to determine the best diagnostic approach.

## 4. Effects of Maintenance Immunosuppression in Diabetic Kidney Disease

### 4.1. Calcineurin Inhibitors

One of the mainstay therapies to prevent allograft rejection involves calcineurin inhibitors (CNIs). CNIs bind to a group of cytoplasmic receptors, including cyclophilin for cyclosporin and FK binding protein for tacrolimus. This drug–receptor interaction inhibits calcineurin activity, which inhibits the transcription of interleukin-2 and several other cytokines in T lymphocytes [22]. Cyclosporin and tacrolimus inhibit human insulin promoter–reporter gene expression, thus interfering in the stimulation of insulin secretion (Table 1). One of the mechanisms of the diabetogenic effect of CNIs includes the inhibitory effects on insulin gene transcription in normal islets cells, which appears to be clinically significant given the high potency of both cyclosporin and tacrolimus in the islets [23]. Furthermore, CNIs regulate the dephosphorylation of the nuclear factor of activated T-cell protein. The dephosphorylation of this protein regulates target genes, which are critical in β-cell survival [24].

Although these agents are widely used in transplantation, both can cause nephrotoxicity and hemolytic–uremic syndrome. Tacrolimus is less likely to cause hyperlipidemia, hypertension, and cosmetic problems but more likely to induce post-transplantation diabetes [23,25]. In a phase III open-label comparative noninferiority study, 638 subjects receiving de novo kidney transplants were randomized to one of three treatment arms: tacrolimus extended-release, tacrolimus, or cyclosporin. HgbA1c levels were collected every six months; the four-year Kaplan–Meier estimate for incidence of HgbA1c levels ≥ 6.5% was significantly higher for both tacrolimus formulations compared to cyclosporin [24].

Understanding the pathophysiology of PTDM in the setting of CNI use can lead us toward potential preventive approaches. A recent study investigating human islets transplanted into immunodeficient mice treated with tacrolimus or sirolimus at clinically relevant levels demonstrated increased amyloid deposition and disruption of islet macrophages’ insulin granule formation, and induced broad transcriptional dysregulation related to peptide processing, ion/calcium flux, and the extracellular matrix. Interestingly, the β-cell abnormalities reversed upon withdrawal of drug treatment. Furthermore, co-treatment with a GLP-1 receptor agonist prevented tacrolimus-induced β-cell dysfunction and partially prevented sirolimus-induced β-cell dysfunction [26].

### 4.2. Glucocorticoids

Glucocorticoids are potent anti-inflammatory agents that play a crucial role in preventing organ rejection. Glucocorticoids are a risk factor for hyperglycemia and diabetes mellitus [27]. The mechanisms of action by which glucocorticoids cause hyperglycemia are increased insulin resistance in the skeletal muscle, causing reduced glucose uptake and reduced glycogen synthesis [28], induced oxidative stress, and release of cytochrome c and suppression of survival factors, leading to apoptotic β-cell death apoptosis [29]. The incidence of hyperglycemia increases with the dose and duration of glucocorticoid therapy. However, a plateau effect might be observed at a specific dose of glucocorticoids, after which there is no worsening hyperglycemia [30].

The dosages and duration of glucocorticoid use can vary among transplant centers and individual patients. Some centers have adopted steroid-sparing protocols to minimize glucocorticoid exposure, while others continue to use them as a cornerstone of immunosuppression. Steroid minimization is a strategy to diminish the risk of PTDM. However, the data supporting this strategyneeds to be more compelling. A study comparing outcomes with early corticosteroid withdrawal and chronic low-dose corticosteroid therapy, including 386 patients, found no difference in the incidence of new-onset diabetes after transplant [31]. Furthermore, a meta-analysis of corticosteroid withdrawal between three and six months after transplantation found no effect on PTDM incidence [32]. There is an increased risk of acute rejection with corticosteroid-sparing strategies [31], and the degree of glycemic burden from low-dose corticosteroid maintenance therapy is unclear; thus, as clinicians, we should consider the risks versus the benefits of such an approach.

### 4.3. mTOR Inhibitors

Another potent class of immunosuppressive agents commonly employed as maintenance therapy in renal transplantation are the mammalian target of rapamycin inhibitors (mTOR-Is), including sirolimus and everolimus [33]. Their main mechanism of action includes inhibiting the mTOR-mediated signal transduction pathways, thereby blocking the cell cycle progression from the G1 to the S phase and cellular proliferation, resulting in inhibition of T- and B-lymphocyte activity [33]. While these agents are often utilized as alternatives to CNIs to reduce nephrotoxic effects or preferentially used for their antioncogenic properties [34], they have the potential to increase the risk of metabolic adverse effects such as hypercholesterolemia, hypertriglyceridemia, and PTDM [33,35].

Several studies have suggested an association between mTOR-Is and diabetes mellitus. Johnston et al. observed that sirolimus was independently associated with new-onset diabetes, whether combined with cyclosporin, tacrolimus, or antimetabolic [36]. Romagnoli et al. retrospectively noted that the incidence of PTDM was significantly higher among kidney transplant recipients with sirolimus and cyclosporin versus cyclosporin monotherapy (31.6% vs. 10.4% *p* = 0.0144, odds ratio 3.97) [37]. In a 10-year retrospective analysis, Gyurus et al. found that immunosuppressive regimens that included sirolimus were more likely to develop diabetes after kidney transplantation [38].

Possible proposed mechanisms from in vitro and in vivo studies suggest that mTOR-Is may decrease β-cell mass through apoptosis [39,40,41,42], impair glucose-dependent insulin secretion [43,44], and increase glucose intolerance and insulin resistance [45,46,47]. Furthermore, insulin signal transduction may be one of the main disruptions in glucose metabolism mediated by mTOR-Is. Within the insulin signal transduction pathway, mTOR exists in two multimeric complexes, mTORC1 and mTORC2, where mTORC1 regulates the phosphorylation of p70 ribosomal protein S6 kinase and promotes protein synthesis and mTORC2 plays a role in phosphorylation of Akt [43]. The mTORC1 is activated by insulin and insulin-like growth factors (IGF) through the IRS/PI3K/Akt pathway [48]. With mTOR-Is binding to mTOR, the PI3K/AKT pathway is suppressed, reducing Akt phosphorylation [48]. This inhibition subsequently impairs glucose-stimulated insulin secretion and proinsulin biosynthesis [43].

### 4.4. Azathioprine and Mycophenolic Acid

Azathioprine is an imidazolyl derivative of mercaptopurine; metabolites are incorporated into replicating DNA and halt replication; it also blocks the pathway for purine synthesis. The principal side effect of azathioprine is dose-related bone marrow suppression [49]. Mycophenolate mofetil and mycophenolate sodium are converted in the liver to mycophenolic acid, the active compound. The target of mycophenolic acid is inosine monophosphate dehydrogenase, the rate-limiting enzyme in the de novo synthesis of guanosine nucleotides, which are essential for DNA synthesis. Blockage of this pathway results in blockage of lymphocyte proliferation [49,50].

The most significant gastrointestinal side effect is diarrhea. It may also cause other gastrointestinal side effects and bone marrow suppression. [50]. In a large study using data from the United Renal Data System, including 11,659 Medicare beneficiaries who received their first kidney transplant in 1996–2000, the investigators found that azathioprine or mycophenolate mofetil reduced the risk of PTDM—relative risk 0.84, 95% CI 0.72–0.97 and 0.78, 95% CI 0.69–0.88, respectively [7].

### 4.5. Belatacept

Belatacept is a human fusion protein combining the extracellular portion of cytotoxic T-lymphocyte-associated antigen 4 with the constant-region fragment (Fc) of human IgG1 (CTLA4Ig) [51]. Belatacept is a selective costimulation blocker that binds surface costimulatory ligands (CD80 and CD86) of antigen-presenting cells. The interaction of CD80 and CD86 with T-cells’ surface costimulatory receptor CD28 is required to activate T-cells fully. Its blockade inhibits T-cell activation [52].

Due to the mechanism of action, belatacept is not associated with cardiometabolic side effects, including PTDM. A landmark trial described similar or slightly lower lipid levels and blood pressure values in the belatacept group compared with the cyclosporin group [53]. On follow up, there was no increased risk of PTDM compared with cyclosporin [54].

Calcineurin-inhibitor-sparing strategies in kidney transplantation are sometimes utilized to minimize the adverse effects in patients of these drugs. A meta-analysis assessing outcomes associated with reducing calcineurin inhibitor exposure from the time of transplantation, including 56 studies comprising data from 11,337 renal transplant recipients, demonstrated that CNI-sparing strategies are associated with decreased new-onset diabetes [55].

## 5. Therapeutic Options for Post-Transplant Diabetes

### 5.1. Insulin

Insulin is an effective pharmacotherapy in the context of high glucocorticoid doses early following transplant. Early and aggressive use of insulin may also have long-term benefits. In a randomized controlled trial, Hecking et al. demonstrated the benefit of early basal insulin therapy following the detection of early post-transplant hyperglycemia at reducing subsequent odds of developing PTDM within the first year following transplantation [56]. Furthermore, an open-label multicenter randomized trial compared 133 kidney transplant recipients given intermediate-acting insulin isophane for postoperative afternoon glucose ≥140 mg/dL with 130 patients given short-acting insulin for fasting glucose ≥200 mg/dL (control). The primary endpoint was PTDM (antidiabetic treatment or oral-glucose-tolerance-test-derived 2 h glucose ≥200 mg/dL) at month 12 post-transplant. The primary endpoint in the intention-to-treat population did show a benefit between treatment and control groups. In the per-protocol analysis, early basal insulin therapy resulted in significantly higher hypoglycemia rates but reduced odds for overt PTDM [57].

### 5.2. Insulin Secretagogues

There are two classes of insulin secretagogues: sulfonylureas and meglitinides. Sulfonylureas lack efficacy and safety data in PTDM but are commonly used due to their rapid efficacy and ease of administration. One pharmacokinetic study evaluating glipizide found that glipizide altered the pharmacokinetics of cyclosporin in renal transplant recipients with PTDM [58]. Due to their prolonged binding time to the β-cell resulting in prolonged insulin secretion, there is an increased risk of hypoglycemia and weight gain [59]. Hypoglycemia risk can increase and prolong renal insufficiency [60]. Although structurally different from sulfonylureas, meglitinides work similarly to stimulate insulin secretion. In a small observational trial, Turk et al., repaglinide was efficacious in lowering mean HbA1c from 7.6 ± 0.6% to 5.8 ± 0.6% after six months in kidney transplant recipients with PTDM [61]. Similarly, meglitinides are associated with increased risk of hypoglycemia in advanced CKD [62].

### 5.3. Biguanides

Metformin is an oral antidiabetic drug in the biguanide class. This medication is contraindicated in patients with advanced kidney disease due to its increased risk of lactic acidosis [63]. A large retrospective study in the United States aiming to determine the frequency of metformin use and its associations among kidney transplant recipients found no association with worse patient or allograft survival [64]. A small randomized trial including 19 patients found similar tolerability and feasibility between metformin and standard of care [65]. Even with some evidence supporting the safety of metformin among kidney transplant recipients, there is still not enough evidence showing benefits among these populations.

### 5.4. Thiazolidinediones

Some data suggest that thiazolidinediones are safe and can potentially benefit patients with PTDM. Most studies are small case series with similar results (Table 2). The largest study—a double-blind randomized controlled trial by Kharazmkia et al. comparing pioglitazone plus insulin versus placebo plus insulin—found a decrease in blood in HgbA1C among those kidney transplant recipients on pioglitazone and no effect on cyclosporin levels [66].

### 5.5. Dipeptidyl Peptidase 4 Inhibitors

There isdata supporting DPP-4 inhibitors among patients with a kidney transplant (Table 2). A pilot study included 13 patients with a kidney transplant, evaluating the effects of sitagliptin in the sirolimus and tacrolimus levels and changes in kidney function. This study found no significant change in three months in sirolimus and tacrolimus levels or kidney function [67]. In 2014, Haidinger et al. conducted a double-blind randomized controlled trial in a single center; the primary outcome was a change in oral glucose tolerance test from baseline to three months. The investigators found a statistically significant reduction in the two-hour plasma glucose (−73.7 ± 51.3 mg/dL vs. −5.7 ± 41.4 mg/dL, *p* ≤ 0.01). Furthermore, there were no severe adverse events or drug interactions with the immunosuppressants [69]. In 2020, Abdelazis et al. investigated the difference in Hgb A1C and graft function among those patients treated with DPP-4 inhibitors versus placebo or another hypoglycemic agent. There was a significant favorable glycemic effect among those treated with DPP-4 inhibitors and no significant change in graft function and tacrolimus level [72].

### 5.6. SGLT-2 Inhibitors

Sodium–glucose transport SGLT2 inhibitors are an essential class of medications that reduce rates of hyperglycemia by promoting urine glucose excretion via blocking the sodium–glucose cotransport proximal tubular cells. This mechanism results in osmotic diuresis, which drags tubular fluid and urine glucose excretion. Volume depletion, increased risk of genital mycotic infections, and euglycemic ketoacidosis can result from this.

SGLT2 inhibitors are effective in slowing chronic kidney disease progression in patients with kidney disease. The CREDENCE trial demonstrated the benefit of SGLT2 inhibitors to decrease the risk of kidney failure and cardiovascular events among persons with type 2 diabetes and albuminuric chronic kidney disease [95]. Other relevant randomized controlled trials, such as the EMPA-REG OUTCOME trial, CANVAS, and DECLARE-TIMI-58, have also demonstrated improvement in kidney and cardiovascular outcomes in patients with kidney disease [96,97,98]. These findings are validated in a recently published study, the EMPA-KIDNEY trial, which demonstrated a reduction in the progression of kidney disease of 0.71 (95% CI, 0.62 to 0.81) [99].

Although the effectiveness of SGLT2 inhibitors in kidney transplantation has not been well-studied, several studies demonstrate their safety and effectiveness (Table 2). Multiple observational studies showed a significant reduction in HbA1c and weight without affecting the allograft function and immunosuppressant levels [85,86,87,88,89,90,91,92,93]. Halden et al. demonstrated a significant reduction in HbA1c with empagliflozin vs. placebo of −0.2% (−0.6, −0.1) vs. −0.1% (−0.1, 0.4), *p* = 0.025 and a significant reduction in body weight of −2.5 kg (−4.0, −0.05) with no difference in adverse events, immunosuppressive drug levels, and allograft function [86]. There is a small but significant increase in the risk of urinary tract infections with SGLT2 inhibitors, which is of particular concern in kidney transplantation patients. None of these studies reported patients with urosepsis pyelonephritis, and there is evidence that the incidence of cystitis is comparable with non-kidney-transplant patients who use SGLT2 inhibitors. More quality evidence is needed, but SGLT2 inhibitors show promising evidence in post-transplant patients.

### 5.7. Glucagon-Like Peptide 1 Receptor Agonists (GLP1- RA) and Glucose-Dependent Insulinotropic Polypeptide-Glucagon-Like Peptide 1 Receptor Agonists (GIP-GLP1-RA)

Incretin hormones comprise two primary hormones, the glucose-dependent insulinotropic polypeptide (GIP) and glucagon-like peptide 1 (GLP-1), produced by the intestinal mucosa in response to oral food intake, playing a vital role in the modulation of glucose metabolism [100]. GLP-1 RA and GIP-GLP-1RA are now widely utilized in managing diabetes in type 2 diabetes mellitus. GLP-1 RA stimulates insulin secretion in a glucose-dependent manner while inhibiting glucagon secretion, increasing satiety and delaying gastric emptying [100]. Like GLP-1 RA, GIP-GLP1-RA enhances insulin secretion and reduces glucagon in a glucose-dependent manner while also increasing insulin sensitivity and slowing gastric emptying [100]. Given the glucose-dependent mechanisms, the risk of hypoglycemia is low, except when used with other antihyperglycemic agents such as insulins or sulfonylureas [101]. Reduction in HbA1c with this class is estimated to be approximately 1–2%, and weight loss between 2 and 6 kg depending on the agents [101].

In type 2 diabetes mellitus, long-acting GLP-1 RAs (dulaglutide, liraglutide, and subcutaneous semaglutide) have demonstrated a reduction in the risk of major adverse cardiovascular events compared to placebo in large cardiovascular outcome trials [102,103,104]. In these trials, kidney endpoints, such as reduced risk of new or worsening nephropathy, were explored as secondary outcomes. The current guidelines recommend GLP-1 RAs with demonstrated cardio-renal benefits to patients with established cardiovascular diseases or multiple cardiovascular risk factors [105,106,107].

The data surrounding GLP-1 agonists in the post-renal transplant setting are limited to small case series and single-center retrospective studies without controls (Table 2). Overall, when used in kidney transplant recipients, the effects on HbA1c, weight, and gastrointestinal side effects appear to be comparable to the general nontransplant population (Table 2). Significant dose reductions were required in those on insulin and concomitant GLP-1 RA therapy. There also appear to be no significant changes in tacrolimus levels and renal function with the addition of GLP-1ra [76,77].

The use of GLP-1 RA is contraindicated in patients with personal or family history of medullary thyroid cancer endocrine neoplasia syndrome. The most common side effects of GLP-1 RAs are gastrointestinal, including nausea, vomiting, diarrhea, or constipation [100]. Caution may be necessary when initiating these agents in post-kidney recipients to mitigate the risk of gastrointestinal side effects with slow dose escalation and close monitoring of adverse effects.

## 6. Practice Considerations

The initial step to prevent PTDM is to identify those persons at risk of PTDM and provide counseling on potential outcomes. Risk factors associated with PTDM are older age, South Asian or African/Caribbean ethnicity, sedentary lifestyle, overweight or obesity, and strong family history. The next step is to encourage lifestyle modifications prior to transplantation. There are no studies on the effect of pharmaceutical interventions on losing weight prior to transplantation; with the novel GLP-1 agonists, there is potential for pharmaceutical interventions among patients with advanced kidney disease or prior to transplantation.

In the post-transplant period, lifestyle modification has a potential benefit but is still not validated by large clinical trials. In a randomized controlled trial, 130 nondiabetic kidney transplant recipients with stable allograft function were assigned in a 1:1 ratio to receive active intervention (lifestyle advice delivered by renal dietitians using behavior change techniques) versus passive intervention (advice alone). This study demonstrated improvements in secondary clinical outcomes, including weight loss and reduced fat mass incidence; however, there was no improvement in insulin secretion, sensitivity, and disposition index [108].

**Figure 1:**
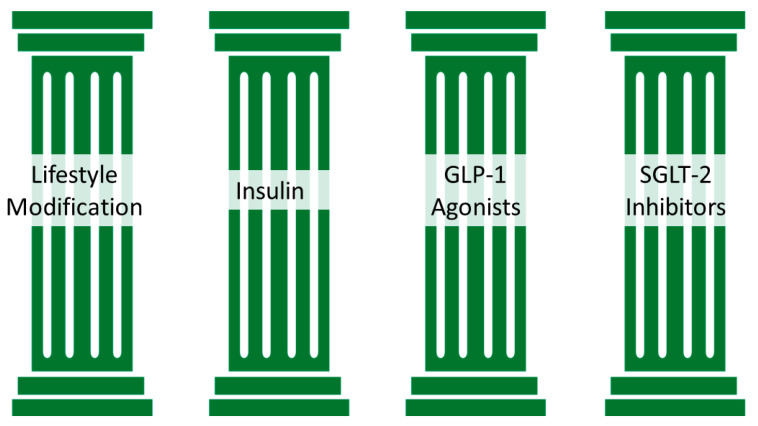
The pillars of PTDM care. Lifestyle modification must be implemented even prior to transplantation; insulin therapy in the early post-transplant period, GLP-1 agonist could be initiated in the outpatient setting early post-transplant and SGLT-2 inhibitors once a baseline creatinine has been established and there are no concerns for urinary tract infections.

In recent years, there have been a series of studies to determine if decreasing or switching immunosuppressive regimens could reduce the risk of PTDM [23,55,109,110]. However, these studies did not show clear evidence that the risk of PTDM can be lowered without increasing the risk of rejection. Given the lack of evidence to make significant changes, we recommend maximizing the immunosuppressive regimen per each transplant center without making changes to decrease the risk of PTDM. Insulin is the safest and most effective option to treat hyperglycemia in the immediate post-transplant period. There are data supporting early insulin use to prevent PTDM [56]. Oral antihyperglycemics like insulin secretagogues may be considered in the early post-transplant period. However, their use must be balanced with the risk of hypoglycemia, particularly when immunosuppressive regimens are being adjusted and titrated downwards to minimize the risk of adverse effects. There are data supporting the use of SGLT2 inhibitors among kidney transplant recipients; observational studies showed a significant reduction in HbA1c and weight. More evidence exists on the kidney and cardiovascular benefits of novel antiglycemic medications. Among persons with type 2 diabetes mellitus, the long-acting GLP-1 agonists are associated with a reduced risk of major adverse cardiovascular events compared to placebo. Data supporting GLP-1 benefits among transplant recipients are needed; however, there are no significant side effects such as infections; therefore, its use must be considered in patients with PTDM. Based on the most recent data and lack of significant side effects, we recommend this novel approach to treat PTDM: (1) lifestyle modification, (2) insulin, (3) SGLT2 inhibitors, and (4) GLP-1 agonists (Figure 1). Although metformin is considered first-line therapy, the novel antidiabetic medications have more cardiovascular benefits than metformin. Thus, we encourage novel antidiabetic use in the post-transplant population, which is almost always affected by cardiovascular disease.

PTDM presents a significant challenge for both patients and healthcare professionals. Research in this field needs to advance, and understanding the association between PTDM and outcomes is important. Studies exploring strategies to prevent and manage PTDM to improve transplant recipients’ long-term outcomes are needed. A deeper understanding of the long-term benefits of the novel antidiabetic medications is encouraged.

## Figures and Tables

**Table 1:** Immunosuppression and its effects on diabetes.

Drug Type	Pathophysiology
mTOR inhibitors	Increase in apoptosis Decrease in β-cell size Reduction in basal and insulin-stimulated glucose uptake and glycogen synthesis Reduction in basal and insulin-stimulated glucose uptake and glycogen synthesis Decrease in insulin-stimulated Akt phosphorylation
Calcineurin Inhibitors	Both tacrolimus and cyclosporin have diabetogenic effects Decrease in insulin secretion Increase in insulin resistance Toxicity on β-cells Tacrolimus has more diabetogenic effects than cyclosporin
Mycophenolate	No diabetogenic effect
Belatacept	Not independent diabetogenic effect Decreased risk compared to Tacrolimus
Glucocorticoids	Increased insulin resistance Increased gluconeogenesis Suppressed insulin secretion Β-cell apoptosis

**Table 2:** Agents and studies in kidney transplant patients.

Study	Methods	Conclusions
**Insulin Secretagogues**		
Turk et al., 2006 [61] N = 23	**Study type:** Observational study single-center **Treatment:** Repaglinide versus rosiglitazone (control group) **Primary outcome:** improvement in blood glucose concentration and HbA1c <7% in the absence of glucosuria and without need for additional antidiabetic agents **Follow-up period:** 6 months	14 of 23 patients were successfully treated Mean HbA1c decreased for 7.6 ± 0.6% to 5.8% ± 0.6% Nine patients had persistent hyperglycemia and were switched to insulin treatment HbA1c (8.5 ± 2.9% at the beginning to 7.4 ± 2.2%)
**DPP4 inhibitors**		
Lane et al., 2011 [67] N = 13	**Study type:** Pilot study single-center **Treatment:** Sitagliptin 100 mg **Primary outcome:** Effect of sitagliptin on tacrolimus and sirolimus levels and change in renal function. **Follow-up:** 3 months	No significant change in tacrolimus and sirolimus levels and no change in eGFR (58.9 ± 4.4 mL/min at entry and 60.5 ± 5.6 mL/min at week 12) HbA1c improved from a baseline of 7.2% ± 0.1 to 6.7% ± 0.2 (*p* = 0.002)
Strom et al., 2014 [68] N = 19	**Study type:** Randomized controlled cross-over, single-center **Treatment:** Sitagliptin 50–100 mg vs. sitagliptin-free period **Primary outcome:** Change in oral glucose tolerance test, insulin and laser Doppler flowmetry assessment of endothelial function with sitagliptin **Follow-up:** 4 weeks	The median first- and second-phase insulin increased by 56.3% (45.2–112.6%, *p* = 0.005) and 39.3% (26.5–81.0%, *p* = 0.006). Fasting and 2 h serum glucose fell 0.9 mmol/L (0.5–1.7 mmol/L, *p* = 0.003) and 2.9 mmol/L (0.5–6.4 mmol/L, *p* = 0.004), respectively. No serious adverse events were observed
Haidinger et al., 2014 [69] N = 33	**Study type:** Prospective double-blind, randomized, placebo-controlled phase II trial. Single-center. **Treatment:** Vildagliptin (17), Placebo (16) **Primary Outcome:** Change in OGTT-derived 2HPG from baseline to 3 months **Follow-up:** 16 weeks	Reduction in 2HPG in vildagliptin compared with placebo (−73.7 ± 51.3 mg/dL vs. −5.7 ± 41.4 mg/dL, *p* ≤ 0.01) No severe adverse events were observed. No drug interactions with the immunosuppressants.
Boerner et al., 2014 [70] N = 22	**Study type:** Observational retrospective. Single-center **Treatment:** Sitagliptin alone (19), sitagliptin and other meds (1), sitagliptin stopped and other meds started (2). **Primary outcome:** Efficacy and safety of sitagliptin in terms of diabetes control, side effects, immunosuppressant levels, and graft function. **Follow-up:** 12 months then extended	HbA1c was significantly improved at 6 and 12 months Analysis of long-term follow-up (32.5 ± 17.8 months) revealed that 17/22 patients remained on sitagliptin (mean hemoglobin A1c <7%), with 9/17 patients remaining on sitagliptin alone Transplant-specific adverse events were rare
Bae et al., 2016 [71] N = 65	**Study type:** Observational retrospective longitudinal, single-center **Treatment:** Vildagliptin (N = 17), Sitagliptin (N = 28), and Linagliptin (N = 20) **Primary outcome:** Glucose-lowering efficacy (HbA1c) of the DPP-4 inhibitors and cyclosporin changes with each drug **Follow-up period:** 3 months	Linagliptin demonstrates superior glucose-lowering efficacy (mean HBA1c −1.40 ± 1.34 *p* = 0.016) Cyclosporin trough levels were increased in sitagliptin compared with vildagliptin (30.62 ± 81.70 ng/mL vs. 24.22 ± 53.54 ng/mL *p* = 0.036) in kidney transplant patients
Abdelazis et al., 2020 [72]	**Study type:** Systematic Review and Meta-Analysis **Treatment:** Any DPP-4 inhibitor vs. placebo or other hypoglycemic agent **Primary outcome:** Difference in HbA1c. **Safety endpoints:** worsening of graft functions and change in Tacrolimus trough level.	Favorable glycemic effect in HbA1c (−0.993, 95% CI = −1.303 to −0.683, *p* = 0.001) Not significant change in eGFR (0.147, 95% CI = −0.139–0.433, *p* = 0.312) Not significant change in Tacrolimus level (0.152, 95% CI = −0.172 to 0.477, *p* = 0.354).
**GLP-1 Agonists**		
Liou et al., 2018 [73] (N = 7)	**Study type:** Retrospective single-center **Treatment:** Liraglutide **Primary outcome:** long-term benefits of liraglutide in management of DM in kidney transplant recipients **Follow-up period:** 19.4 ± 7.6 (range 10.5–27.6) months.	Glycemic control improved fasting blood sugar (FBS) from an initial 228.6 ± 39.1 mg/dL to a final FBS of 166.0 ± 26.6 mg/dL (*p* = 0.103), glycated hemoglobin (HbA1c) from an initial 10.0 ± 1.6% to a final 8.1 ± 0.8% (*p* = 0.043). The average body weight was from an initial of 78.0 ± 7.8 kg to a nadir of 75.1 ± 9.1 kg (*p* = 0.032). No significant change in urinary protein to creatinine ratio.
Singh et al., 2019 [74] (N = 63)	**Study type:** Retrospective single-center **Treatment:** Dulaglutide **Primary outcome:** change in weight (kg), BMI (kg/m^2^), insulin requirements (units), Cardiovascular morbidity, graft-survival, and all-cause mortality **Follow-up period:** 6, 12, 24 months	Mean of the paired difference for reduction in weight was 2.07 (*p* value < 0.003), 4.007 (*p* value < 0.001), and 5.235 kg (*p* value < 0.034) and in BMI was 0.808 (*p* value < 0.001), 1.352 (*p* value < 0.005), and 2.015 kg/m^2^ (*p* value < 0.045) at 6, 12, and 24 months, respectively The mean daily insulin requirement before dulaglutide treatment was 22.9 units, which decreased to a mean of 17.03 units after dulaglutide treatment (mean of paired difference, 5.95 units; *p* = 0.0002).
Singh et al., 2020 [75] (N = 88)	**Study type:** Retrospective single-center (US) **Treatment:** Dulaglutide (N = 63), Liraglutide (N = 25) **Primary outcome:** assess the safety and effectiveness of dulaglutide and liraglutide in solid organ transplant with diabetes **Follow-up period:** 6 months, 12 months, 24 months	The % decreases in weight were 2%, 4%, and 5.2% with dulaglutide, and 0.09%, 0.87%, and 0.89% with liraglutide, at 6, 12, and 24 months, respectively. BMI % reductions of 2.4%, 6%, and 8% with dulaglutide, and minimal decreases of 0.24%, 1.4%, and 0.54% with liraglutide, at 6, 12, and 24 months, respectively (*p* values < 0.05 throughout the study period). The % reduction in insulin requirement was 26% with dulaglutide versus 3.6% with liraglutide at the end of follow-up (*p* = 0.01).
Thangavelu et al., 2020 [76] (N = 19)	**Study type:** Retrospective single-center **Treatment:** Liraglutide (N = 10), dulaglutide (N = 5), semaglutide (N = 2), and exenatide (N = 2) **Primary outcome:** changes in immunosuppressant levels, rejection episodes, changes in hemoglobin A1c (HbA1c), weight, and body mass index (BMI) while on the GLP-1RA **Follow-up period:** 12 months	Kidney transplants: 7 patients No significant changes in tacrolimus levels and renal function Mean decrease in weight was 4.86 kg [95% CI −7.79, −1.93] BMI decreased by mean of 1.63 kg/m^2^ at the end of 12 months [95% CI −2.53, −0.73] HbA1c decreased from baseline by 1.08% [95% CI −1.65, −0.51], 0.96% [95% CI −1.68, −0.25], and 0.75% [95% CI −1.55, 0.05] at 3, 6, and 12 months, respectively
Kukla et al., 2020 [77] (N = 17)	**Study type:** Retrospective single-center **Treatment:** Liraglutide (N = 14), dulaglutide (N = 2), and exenatide (N = 1) **Primary outcome:** efficacy, safety, and the effect on kidney allograft function in kidney transplant recipients on GLP1a **Follow-up period:** at least 12 months (N = 14)	Median body weight and BMI at the therapy implementation was 106 [IQR 98.5–125.2] kg and 36.5 [IQR 34.7–38.1] kg/m^2^, respectively. Not statistically significant at 12 months. Reduction in the total daily insulin dose, from the median of 63 [interquartile range 43–113] IU to 44 [interquartile range 25–88], and reduction in the risk of hypoglycemia Median eGFR at therapy initiation was 52 [IQR 40–60] mL/min/1.73 m^2^ and kidney function remained stable. Tacrolimus dose did not require adjustment and was not significantly changed within 4 months of initiation (*p* = 0.3)
Kim et al., 2020 [78] (N = 37)	**Study type:** Retrospective single-center **Treatment:** Dulaglutide (0.75 mg and 1.5 mg) and basal insulin vs. multiple insulin injections **Primary outcome:** efficacy of dulaglutide compared to prandial insulin in kidney transplant recipients with T2DM undergoing multiple daily insulin injection (MDI) therapy **Follow-up period:** 6 months	HbA1c 7.1% vs. 7.0% for dulaglutide and MDI injections, respectively; 95% confidence interval [CI], −0.53 to 0.28; *p* = 0.53) The basal insulin and dulaglutide combination resulted in a reduction in FPG levels by 9.7 mg/dL (95% CI, 2.09 to 41.54; *p* = 0.03), in body weight by 4.9 kg (95% CI, 2.87 to 6.98; <0.001), and in basal insulin dose by 9.52 IU (95% CI, 5.80 to 3.23; *p* < 0.001).
Yugueros González et al., 2021 [79] (N = 15)	**Study type:** Retrospective single-center **Treatment:** Semaglutide (N = 7), liraglutide (N = 4), dulaglutide (N = 2), and empagliflozin (N = 2) **Primary outcome:** efficacy and safety of GLP-1a and/or SGLT2i in kidney transplant recipients **Follow-up period:** 12 months	Median HbA1c at baseline was 6.7 (interquartile range [IQR] = 5.8–8.2) and at 12 months it was 6 (IQR = 5.3-8.1, *p* = 0.96) Mean weight difference at 12 months was a loss of 7.2 ± 6 kg; median body mass index at baseline was 31.2 kg/m^2^ (IQR = 29.7–35.5) and 29.5 kg/m^2^ (IQR = 27.6–31.6, *p* = 0.01) at 12 months
Vigara et al., 2022 [80] (N = 40)	**Study type:** Retrospective single-center **Treatment:** Semaglutide (19), liraglutide (13), and dulaglutide (8) **Primary outcome:** efficacy of GLP-1RA in kidney transplant recipients **Follow-up period:** 6 months (N = 40), 12 months (N = 26)	eGFR improvement (+3.5 mL/min/1.73 m^2^ at 12 months, *p* = 0.030) and a reduction in proteinuria (−59.1 mg/g at 6 months, *p* = 0.009 and −48.5mg/g at 12 months, *p* = 0.021) Body weight was reduced (−2.4 kg at 6 months, *p* = 0.006 and −3 kg at 12 months, *p* = 0.041). HbA1c was also decreased (−9 mmol/mol at 6 months, *p* < 0.001 and −5 mmol/mol at 12 months, *p* = 0.018)
Sweiss et al., 2022 [81] (N = 118)	**Study type:** Retrospective single-center **Treatment:** Dulaglutide (N = 45), liraglutide (N = 36), semaglutide (N = 32), and exenatide ER (N = 5) **Primary outcome:** efficacy and safety of GLP1-RA for DM in SOT transplants for a minimum of 3–12 months **Follow-up period:** 3–12 months	Kidney transplants: 70% A statistically significant difference was observed in median fasting blood glucose and HbA1c at baseline to 3–12-month nadir (8% (7–9) vs. 7% (6–8); *p* < 0.001). Fasting blood glucose (*p* < 0.0001), body weight (*p* < 0.0001), body mass index (*p* = 0.0008), serum creatinine (*p* < 0.0001), and eGFR (*p* < 0.0001) were all found to be statistically significant from baseline to 3 to 12-month nadir.
**Thiazolidinediones**		
Baldwin Jr et al., (2004) [82] N = 18	**Study type:** Case series, single-center **Treatment:** rosiglitazone 4 mg/d, increased to 4 mg twice daily in 8 patients. **Primary outcome:** Changes in serum creatinine, cyclosporin, or tracrolimus. Change in HbA1c. **Follow-up:** 133–718 days (mean 381 days)	No significant changes in serum creatinine, cyclosporin, or tacrolimus. Mean HbA1c improved from 8.1 ± 1.5% to 6.9 ± 1.3%, *p* = 0.01. No patient developed clinically significant peripheral edema or pulmonary congestion
Villanueva et al., (2005) [83] N = 40	**Study type:** Retrospective, single-center **Treatment:** rosiglitazone 4 mg/d increased to BID when needed. **Primary outcome:** Normalization of levels of HbA1c in patients with post-transplant Diabetes. **Follow-up:** 3–12 months (mean 26 weeks)	30% of the patients maintained normal HbA1C levels (5.6 ± 0.8%) with Rosiglitazone alone, 63% required addition of a sulfonylurea. 63% continued 4 mg/d and 37% required an increase to 8 mg/d. Mild edema developed in 13% of patients.
Voytovich et al., (2005) [84] N = 10	**Study type:** Case series, single-center **Treatment:** Rosiglitazone 8mg/d **Primary outcome:** Changes in glucose disposal rate. Change in fasting and 2 h plasma glucose. **Follow-up:** 4 weeks	Improved mean glucose disposal rate (from 6.5 to 9.7 g/kg/min, *p* = 0.02) and a significant decline in fasting and 2 h plasma glucose (from 6.4 to 5.8 mmol/l, *p* = 0.02 and from 14.2 to 10.6 mmol/l, *p* = 0.03, respectively). No changes in plasma tacrolimus and cyclosporin were observed.
Kharazmkia et al., (2014) [66] N = 64	**Study type:** Double-blind randomized placebo-controlled trial, single-center **Treatment:** pioglitazone (30 mg) plus insulin and placebo plus insulin **Primary outcome:** Changes in blood glucose level **Follow-up:** 4 months	No significant change in blood glucose levels. Decrease in HbA1C% (−1.21 ± 1.2, *p* ≤ 0.001) Four dropouts in pioglitazone group (three had mild to moderate lower extremity edema and one insomnia) Did not affect cyclosporin level or doses (−12.1 ± 28.0 vs. −12.41 ± 7.5, *p* = 0.13)
**SGLT2 inhibitors**		
Rajasekeran et al., (2017) [85] N = 10	**Study type:** Case series **Treatment:** Canagliflozin **Primary outcome:** Mean changes in metabolic and hemodynamic parameters **Follow-up:** 80.5 person-months	Change in HbA1c −0.84% (1.2 SD), *p* = 0.07 Change in weight −2.14 kg (2.8 SD), *p* = 0.07 Change in eGFR −4.3 mL 7 min/1.73 m^2^ (12.2 SD), *p* = 0.3
Halden et al., (2019) [86] N = 49	**Study type:** Prospective, randomized double-blind, single-center **Treatment:** Empagliflozin 10 mg, placebo. **Primary outcome:** Change in mean glucose estimated with continuous glucose monitor. Secondary outcome: change in HbA1c, FPG, 2 h plasma glucose, body weight, WHR (waist/hip ratio), visceral fat, blood pressure, and eGFR. **Follow-up:** 24 weeks	Median change in HbA1c significantly reduced with empagliflozin compared with placebo: −0.2% (−0.6, −0.1) vs. −0.1% (−0.1, 0.4), *p* = 0.025. Significant reduction in body weight of −2.5 kg (−4.0, −0.05) compared with an increase of 1 kg in the placebo group (*p* = 0.014) No significant differences in adverse events, immunosuppressive drug levels, or eGFR
Schwaiger et al., (2019) [87] N = 14	**Study type:** Prospective, interventional pilot, single-center study **Treatment:** Empagliflozin 10 mg (initiated after stopping insulin) **Primary outcome:** Change in the 2 h glucose level in the OGTT (Increase <30 mg/dL suggesting noninferiority compared to insulin) **Follow-up:** 14 participants 4 weeks and 8 participants 12 months	2 h glucose increased from 232 ± 82 mg/dL (baseline) to 273 ± 116 mg/dL (4 weeks, *p* = 0.6) and to 251 ± 71 mg/dL (12 months, *p* = 0.41) Reduction in eGFR from 55.6 ± 20.3 to 47.5 ± 15.1 mL/min/1.73 m^2^ (4 weeks, *p* = 0.008) and to 53.5 ± 13.3 (12 months, *p* = 0.93) Bacterial urinary infections occurred in five empagliflozin participants vs. nine matched reference patients (*p* = 0.81)
Shah M et al., (2019) [88] N = 24	**Study type:** Pilot study, single-center **Treatment:** Canagliflozin 100 mg **Primary outcome:** Change in weight, BP, and HbA1c **Follow-up:** 6 months	Mean body weight was 78.6 ± 12.1 kg before and 76.1 ± 11.2 kg 6 months after, *p* < 0.05) Mean systolic and diastolic BP (mmHg) was 142 ± 21 and 81 ± 9 before and 134 ± 17 and 79 ± 8, 6 months after; *p* < 0.05 Decrease in HbA1c from 8.5% ± 1.5 to 7.6 ± 1, *p* < 0.05. No significant change in creatinine
Attallah et al., (2019) [89] N = 8	**Study type:** Case series **Treatment:** empagliflozin **Primary outcome:** change in creatinine, HbA1c, urine protein, and weight **Follow-up:** 1 year	Urine protein decreased by 0.6 g/d, HbA1c decreased by 0.85% and weight decreased by 2.4 kg During the first month, serum creatinine increased by 12.4% but then stabilized Two patients developed UTI
Mahling M et al., (2019) [90] N = 10	**Study type:** Prospective observational study, single-center **Treatment:** Empagliflozin **Primary outcome:** Change in eGFR, HbA1c Follow up: 6.3 person-years	The median eGFR at baseline was 57 mL/min/1.73 m and remained stable Median HbA1c decreased from 7.3 to 7.1% Two patients developed UTI
AlKindi F et al., (2019) [91] N = 8	**Study type:** Retrospective observational single-center **Treatment:** empagliflozin 10 mg/d (5), empagliflozin 25 mg/d (1) and dapagliflozin 5 mg/d (2) **Primary Outcome:** Changes in HbA1c, renal function, blood pressure, and weight **Follow-up:** 12 months	Decrease in HbA1c from 9.34 ± 1.36 to 7.41 ± 1.44, *p* ≤ 0.05 Decrease in body mass index (BMI) from 32.74 ± 7.2 to 27.4 ± 4.2, *p* ≤ 0.05 No significant change in eGFR, serum creatinine, and blood pressure One patient developed UTI
Lim et al., (2022) [92] N = 2083	**Study type:** Multicenter cohort, propensity score matching **Treatment:** SGLT2 inhibitors vs. non SGLT2 inhibitors **Primary outcome:** Composite outcome of all-cause mortality, death-censored graft failure (DCGF), serum creatinine doubling. **Follow-up:** 62.9 ± 42.2 months	SGLT2i group had a lower risk of primary composite outcome than control group (adjusted HR, 0.43; 95% CI, 0.24–0.78; *p* = 0.006) Decreased risk of DCGF and serum creatinine doubling in the SGLT2i group Overall eGFR remained stable without an initial dip after SGLT2i use
Sweiss H et al., (2023) [93] N = 49 (18 kidney)	**Study type:** Retrospective **Treatment:** SGLT2 inhibitors (26 liver, 18 kidney, 4 lung, and 1 simultaneous liver–kidney recipient) Primary outcomes: included change in hemoglobin A1c, fasting blood glucose, actual body weight, and body mass index. Safety outcomes included adverse effects, cardiovascular events, death-censored graft loss, and all-cause mortality. **Follow-up:** 12 months	Glycemic and weight loss outcomes showed a statistically significant benefit from SGLT2i use Total safety outcome incidence was minimal at 12 months No patient experienced myocardial infarctions, graft loss, or mortality at 3–12 months One incidence of urinary tract infection and stroke occurred each
Lin Y et al., (2023) [94] N = 2417	**Study type:** Systematic Review **Treatment:** Included all primary interventional and observational studies on SGLT2 inhibitors in transplant patients (15 studies total) **Primary outcomes:** Mortality, cardiovascular and kidney events, graft rejection Follow-up: 0.4 to 5.2 years	Could not confirm clinical cardiovascular and kidney benefits in the transplant population SGLT2 inhibitors may improve glycemic control; however, they are associated with increased risk of UTIs Benefit of SGLT2 inhibitors may not outweigh potential harm in solid organ transplant population.

## Data Availability

No data was used to create this review.
